# Trapping and killing performance of a PermaNet 2.0 hybrid mosquito trapping bednet: an experimental hut evaluation

**DOI:** 10.12688/wellcomeopenres.19759.3

**Published:** 2024-03-26

**Authors:** Chouaibou Seidou Mouhamadou, France-Paraudie A. Kouadio, Christabelle G. Sadia, Fodjo K. Behi

**Affiliations:** 1 Centre Suisse de Recherches Scientifiques en Côte d’Ivoire, Abidjan, Cote d'Ivoire; 2 Nangui Abrogoua University, Abidjan, Lagunes Region, Cote d'Ivoire

**Keywords:** Malaria Vector Control, Insecticide Resistance Management, Mosquito Trapping Long Lasting Insecticidal Bednet

## Abstract

**Background:**

Despite the huge global effort , there has been an increase in malaria morbidity and mortality in sub-Saharan Africa since 2015, from 212 million cases and 429,000 deaths in 2015 to 241 million cases and 627,000 deaths in 2020 mainly because of resistance to insecticide. Therefore, advancing innovative approaches is the only sustainable way to fight malaria.

**Methods:**

Taking advantage of the behavior of mosquitoes around the net, which is almost 70-90% concentrated on the roof, we have developed a two-compartment mosquito bednet, the so-called T-Net for mass mosquito trapping and killing. In the current study, we investigated in an experimental hut trial, the efficacy of trapping-long-lasting insecticide-treated nets (T-LLINs) against *Anopheles gambiae* s.l. in an insecticide resistance context. Five different arms have been considered in this study including three positive control arms e.g. PermaNet 2.0 LLIN, Tsara boost LLIN and Interceptor generation 2 (IG2) LLIN), one negative control arm using insecticide-free bednet, and one candidate arm using a hybrid-treated trapping bednet made with PermaNet 2.0 LLIN mounted with an insecticide-free compartment (T-LLIN).

**Results:**

The highest average daily mortality was recorded with the T-LLIN. In total, 678 mosquitoes were killed by T-LLIN among the 760 collected, i.e. 89.2%. Out of these, 317 were found in the trap compartment, representing 46.75% of mortality directly attributable to the mechanical effect of this net. This added value made it possible to quantify the increased in the killing effect that this net would have over the positive control arms: this would be 58.5% higher than the killing effect of PN2.0, 38% higher than that of Tsara boost and 31.5% higher than that of IG2.

**Conclusion:**

The current study shows potential to maximize the efficiency of the WHO-recommended LLINs by an addition of an insecticide-free trap compartment on top of the net.

## Background

Vector control remains the main preventive measure used against malaria. It is based on the use of insecticides in the form of indoor residual spraying or impregnated to mosquito nets for a long duration of action. Over the past two decades, increased funding, intensification of effective interventions and political commitment have made it possible to significantly reduce the global burden of malaria. It is estimated that 663 million cases of malaria have been prevented in sub-Saharan Africa since 2001 through the scaling-up of these control interventions, 69% of which are the direct result of the use of long-lasting insecticide-treated nets (LLINs) ^1^. The susceptibility of mosquito vectors to insecticides determines the effectiveness of insecticide-based malaria control measures. In other words, if vectors become less susceptible by becoming resistant to the toxic effects of insecticides through natural selection and mutations, then interventions based on these compounds, and therefore malaria prevention, become compromised. To date, delaying the development of resistance to insecticides is the only resistance management strategy. It is essentially based on the use of insecticides from different classes and different modes of action, in combination or in rotation in time and space, in order to reduce the selection pressure on the different resistance mechanisms ^2^. However, the limited number of available molecules, cross-resistance and redundancy between insecticide families used in agriculture and public health limit the impact and effectiveness of these management measures. As a result, regardless of the insecticide-based vector control approach, development and dramatic increases in resistance are observed ^3^. The phenomenon has spread to the continent's main malaria vector populations in just a few decades. Malaria morbidity and mortality in sub-Saharan Africa has increased again since 2015, from 212 million cases and 429 000 deaths in 2015 to 241 million cases and 627 000 deaths in 2020 ^4– 8^. Consequently, failure to mitigate the development of new insecticide resistance and manage existing ones can result in an even more increase of disease burden, potentially reversing some of the substantial gains made in controlling malaria over the last decade. Therefore, advancing innovative approaches is the only sustainable way to fight malaria. From this perspective, we hypothesized that adding a mechanical killing mechanism, which targets vectors of all species and resistance status, to conventional LLINs could be a major tool in insecticide resistance management and control of malaria transmission. Taking advantage of the behavior of mosquitoes around the net, which is almost 70–90% concentrated on the roof ^9– 11^, we have developed a two-compartment mosquito bednet, the so-called trapping-net (T-Net) for mass mosquito trapping and killing. Basically, the T-Net is a bednet comprising a lower compartment that serves as a protected sleeping environment for the user, and an upper compartment that serves as a trap for mosquitoes attempting to reach the sleeper. The two compartments being separated by an insecticide-free or -treated roof of the lower compartment ^12^. Indeed, when using a bednet, the sleeper emits a combination of olfactory, chemical, thermal, and visual stimuli ^13^ which attract mosquitoes into the trap compartment through four funnel-like openings which also prevents mosquitoes from escaping once trapped. Trapped mosquitoes die a few hours later of either insecticide, starvation, or desiccation.

In our previous study ^12^, we demonstrated, using an insecticide-free T-Net, that the trapping bednet concept for the capture of aggressive mosquitoes has considerable potential. In the current study, we investigated in an experimental hut trial, the efficacy of trapping-LLINs (T-LLINs). The T-LLIN consists of an insecticide-free trap compartment mounted on a long-lasting mosquito net (LLIN). Details on trap compartment are provided on Mouhamadou *et al.*, ^12^. Our aim was to assess the efficiency of the T-LLIN on mosquito mortality, compared to second-generation LLINs recommended by the WHO in a context of insecticide resistance.

Our ultimate objective is to address the major public health concern of insecticide resistance in malaria vectors, by proposing an innovative simple and cheap way to improve the performance of insecticide-treated nets (ITNs), to be efficient against all mosquitoes regardless of the status of insecticide resistance.

## Methods

The study was conducted in experimental huts according to WHO protocol ^14^ in the municipality of Sakassou (7°27′N 5°17′W) in May-June 2023. Sakassou is a town in central Ivory Coast in in Gbêkê Region. The average temperature in Sakassou is 26.1°C. Rainfall is recorded all year round, with an average annual precipitation of 899.6mm. The malaria transmission is mainly due to *Anopheles gambiae sl* which has developed resistance to insecticides ^15^.

The experimental field station of Sakassou located around 500 meters from a large rice-growing area is composed of 12 WHO-standard West-African type huts ^14^ slightly modified to not include the verandah trap. Entry of mosquitoes is facilitated through four window slits located on three sides of the hut. The slits are designed in such a way as to prevent mosquitoes from escaping once they are inside the hut. Each hut is equipped with two window traps located on the sides to capture mosquitoes that would otherwise escape.

Ten sleepers were trained and paid to sleep in 10 huts under the bednets. Trapping was conducted from 21:00 to 5:00, and the mosquitoes collected each morning after the sleep cycle was completed. Sleepers were rotated on a daily basis while bednets were rotated every three days according to Latin-square tables ^14^ such a way that each bednet goes through each of the ten huts. The trial was conducted for six weeks. Prior to the trial, blank mosquito collection has been carried out to check for any hut’s effect. Sleepers were all vaccinated against yellow fever and were taken to the hospital to check for malaria parasites. The study received the national ethical approval (N/ref:148-22/MSHPCMU/CNESVS-kp), and all sleepers gave written informed consent prior to enrolment in the study. Within the hut, resting and dead non-trapped mosquitoes were collected from the room using 5 mL (12 mm × 75 mm) glass hemolysis tubes. Trapped mosquitoes were removed with mouth aspirators from the T-LLIN trap compartment by the field technicians. All mosquitoes were taken to the field laboratory and identified to genus level and scored as trapped, not trapped-dead, or not trapped-alive ^13^. Live mosquitoes were placed in cups and given access to a sugar solution for 24–36 h in the insectary at 25–27 °C and 70–80% relative humidity to assess delayed mortality.

Five different arms have been considered in this study including three positive control arms e.g. PermaNet 2.0 LLIN, Tsara boost LLIN and Interceptor generation 2 (IG2) LLIN), one negative control arm using insecticide-free bednet, and one candidate arm using a hybrid-treated T-Net made with PermaNet 2.0 LLIN mounted with an insecticide-free compartment. The PermaNet 2.0 used was 100 Dernier treated with 56 mg/m2 of deltamethrin, while Tsara boost used was 130 Dernier treated with 120 mg/m2 of deltamethrin + 440 mg/m2 piperonyl-butoxide (PBO) and the IG2 was 100D treated with 150 mg for chlorfenapyr + 75 mg of alphacypermethrin. Each arm consisted of 2 replicates. All the LLINs are WHO-recommended and were new, made of polyester and provided by the National Malaria Control program in Côte d’Ivoire. Tsara boost and IG2 are considered as WHO gold standards in insecticide-resistant areas in malaria vectors.

In general, four entomological parameters are considered in experimental hut trials ^14^, including the deterrence in the mosquito entry due to candidate net, the exit rate, the blood feeding inhibition, and mortality induced by the candidate net. These parameters are very important when evaluating a novel chemical or chemical combination. However, since the T-LLIN we used is composed by a deltametrin-treated section (PermaNet 2.0) already widely evaluated in experimental hut trials ^16– 18^, we only focused on the mosquito death in our evaluation as this is the primary point of adding a trap compartment. We defined mortality as death due to insecticide (immediate and delayed) + trapped mosquitoes (which were considered dead even if they were alive).

The non-parametric Kruskal-Walis test with Conover-Iman multiple pairwise comparisons and Bonferroni correction was used to compare the mean entry rate between huts, while the Wilcoxon non-parametric test was used for two-by-two comparisons between the T-LLIN and each of the study arm (alpha = 0.05). The analyses were conducted using the XLSTAT software package version 2023.1.6.1410 ^19^. The mortality was used to estimate the increase killing effect of T-LLIN over other LLINs tested as follow: Killing effect (%) = 100 x (K _T-LLIN _– K _LLINs_) / T _LLINs_, where K _T-LLIN_ is the number of mosquitoes killed in the huts with T-LLIN, K _LLINs_ is the number of mosquitoes killed in the huts with the LLINs, and T _LLINs_, is the total number of mosquitoes collected from the huts with LLINs.

## Results

A total of 7835 mosquitoes of all species were collected during the six weeks evaluation, of which 3041 were *An. gambiae*, i.e. 38.8%. Among the other species, direct mortality observed with PN2.0 T-LLIN was 583 deaths out of 1150 mosquitoes collected, i.e. 50.7%. Of the 583 deaths, 323 were mechanical kills, i.e. captured by the trap on the net. This corresponds to 55.4% of total mortality and represents the added value of trapping over the total mortality caused by this T-LLIN.

**Figure 1.**
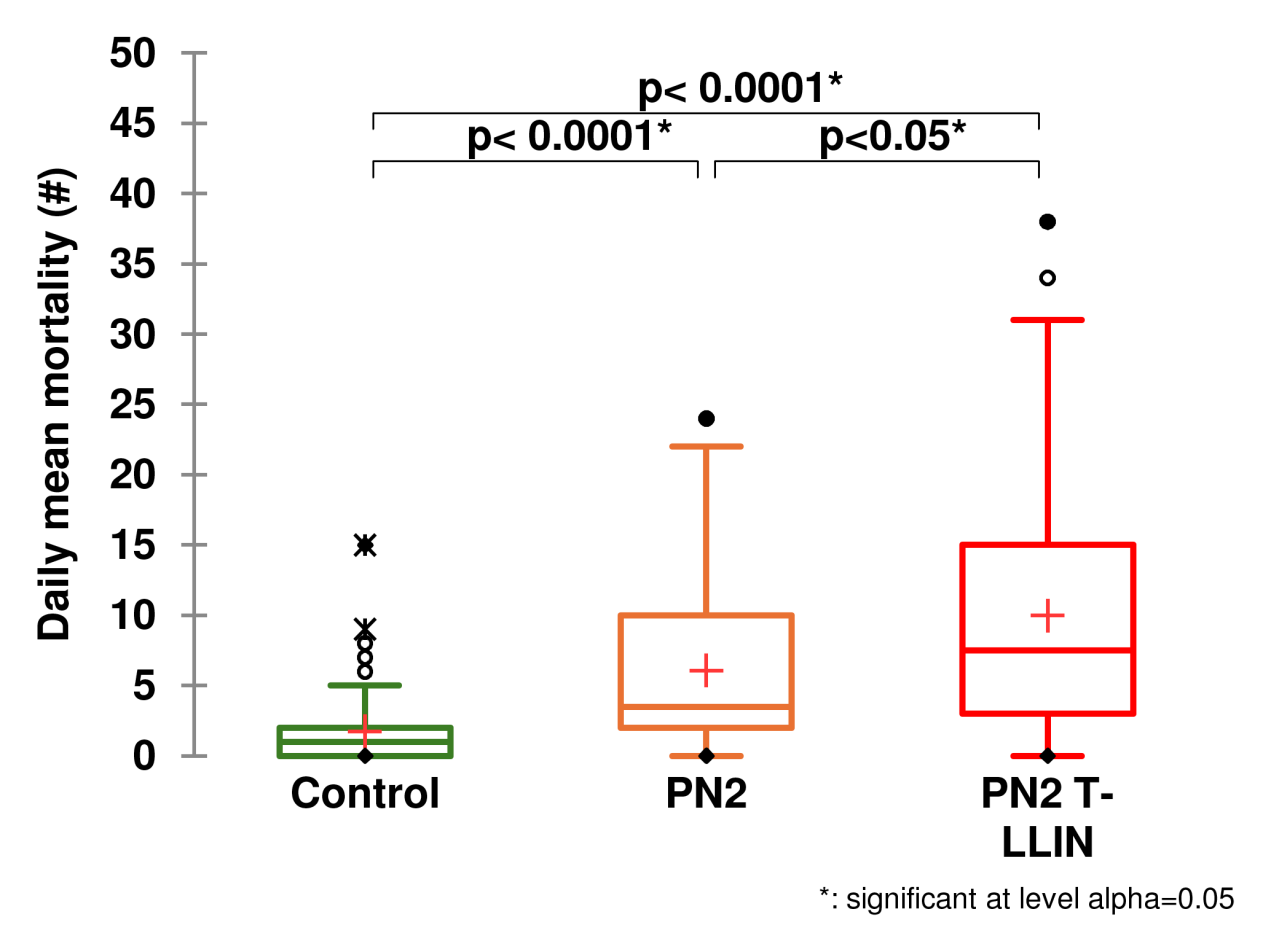
Average number of mosquitoes killed per day for the PN2 T-LLIN compared with PN2.0 only pyrethroid LLIN.

**Figure 2.**
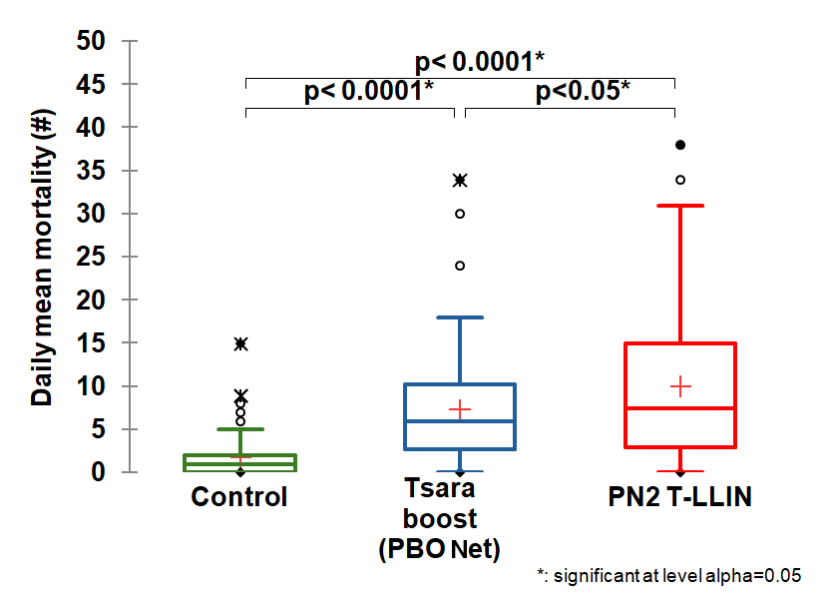
Average number of mosquitoes killed per day for the PN2 T-LLIN compared with Tsara boost PBO-pyrethroid LLIN.

**Figure 3.**
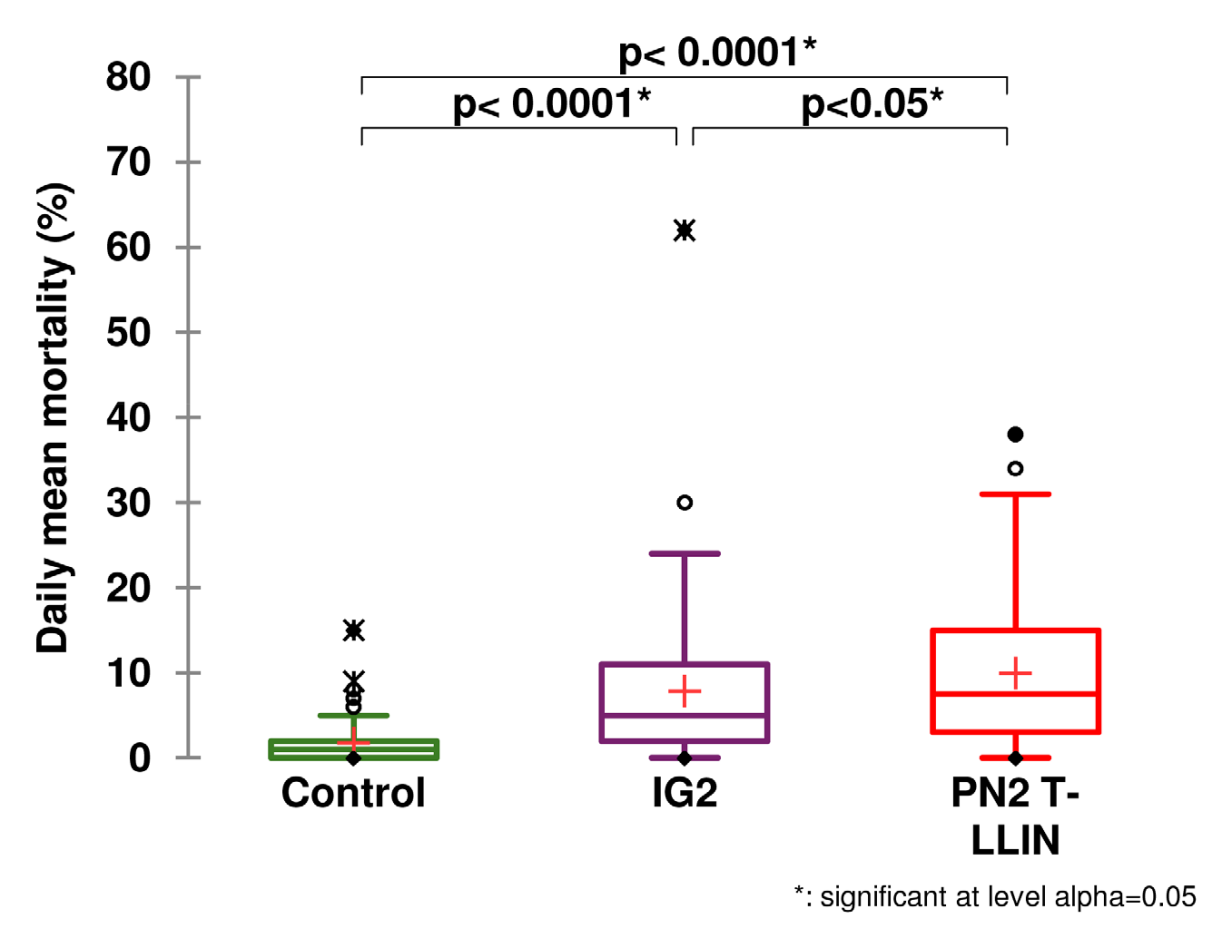
Average number of mosquitoes killed per day for the PN2 T-LLIN compared with IG2 pyrrole-pyrethroid LLIN.

**Figure 4.**
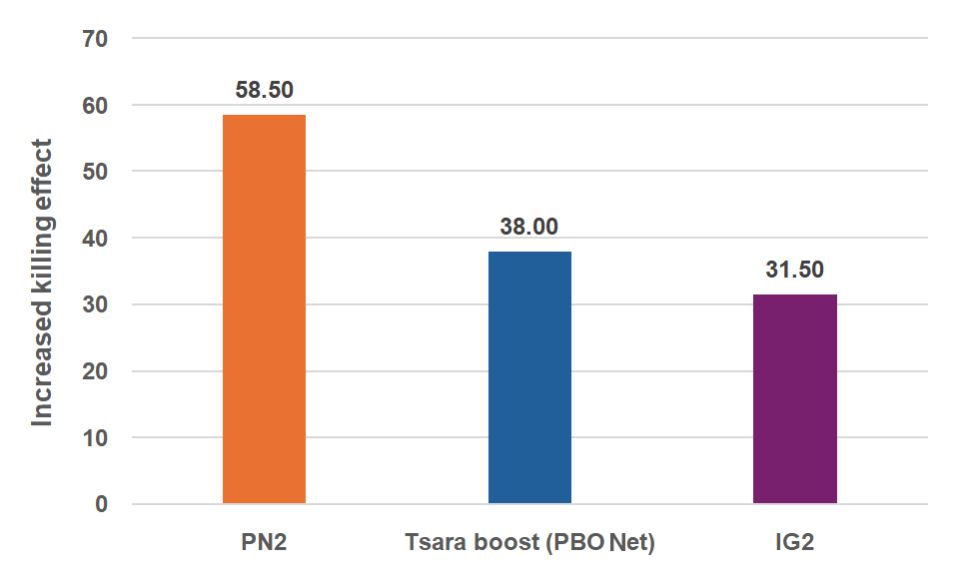
Increased killing effect of T-LLIN (PN2.0-Trapping bednet) over PN2.0, Tsara boost (PBO net) and IG2. The increased killing effect of T-LLIN over other LLINs tested was estimated as follow: Killing effect (%) = 100 x [(K _T-LLIN _– K _Un_) - (K _LLIN_ – K _Un_)] / T _Un_, where K _T-LLIN_ is the number of mosquitoes killed in the huts with T-LLIN, K _LLIN_ is the number of mosquitoes killed in the huts with the LLIN, K _Un LLIN_ is the number of mosquitoes killed in the untreated huts and T _Un_, is the total number of mosquitoes collected from the huts with untreated net.

Concerning *An. gambiae*, Table 1 gives details of the entry rate and mortality obtained in each study arm. Low entry rate and low mortality were observed in the negative control treatment (118 dead /450 collected), i.e. 26.2% with a daily average of 1.7 mosquitoes killed. When we look at the results for our candidate arm compared to the three positive control net arms, we see that the highest average daily mortality was recorded with the T-LLIN ( Table 1, Figure 1, Figure 2, Figure 3). The average number of mosquitoes killed per day was 10 for the PN2 T-LLIN compared with 6.1 for PN2.0 LLIN ( Figure 1), 7.4 for Tsara boost LLIN ( Figure 2) and 7.9 for IG2 LLIN ( Figure 3). In total, 678 mosquitoes were killed by T-LLIN among the 760 collected, i.e. 89.2%. Out of these, 317 were found in the trap compartment, representing 46.75% of mortality directly attributable to the mechanical effect of this net. The mean daily trapping number was 4.7 mosquitoes per day out of 11.2 mean daily entry ( Table 1). This added value made it possible to quantify the increased in the killing effect that this net would have over the positive control arms: this would be 58.5% higher than the killing effect of PN2.0, 38% higher than that of Tsara boost and 31.5% higher than that of IG2 ( Figure 4).

**Table 1.** *Anopheles gambiae* entry rate and mortality obtained in each study arm during experimental hut trial assessing the efficacy of a PermaNet 2.0 trapping bednet compared to IG2, Tsara boost (a PBO net) and PermaNet 2.0 LLINs.

Variable	Total Entry	Mean Daily Entry	Std. deviation	Total Death	Mean Daily Death	Std. deviation
IG2	611	9.0a	11.5	535	7.9a	10.0
Tsara boost (PBO net)	698	10.3a	13.8	505	7.4 a,b	6.7
PN2.0	509	7.5a	7.1	413	6.1 a,b,c	6.0
PN2 T-LLIN	760	11.2a	10.6	678	10.0 d	9.0
Negative Control	450	6.6a	6.6	118	1.7 e	2.7

*Data not sharing the same letters in the same column are statistically significant (P<0.05).

## Discussion/conclusion

The present study investigates the trapping and killing potentials of a PermaNet 2.0 LLIN mounted with a free-insecticide mosquito trapping compartment. It provides further evidence supporting the trapping bednet concept already described in our previous study ^12^ by demonstrating that addition of a mosquito trapping compartment to a LLIN can significantly enhance its effectiveness. The major advantage of this approach is that it is based on the traditional practice of using LLINs, which does not imply the need for a change in the nature of protective measures already well known and integrated in communities. The trapping feature added to the LLIN strengthens the effectiveness of this tool and which should complement the arsenal of malaria control tools currently available to national malaria control programs, while also helping to manage populations of resistant mosquitoes and, potentially, improving the acceptability and hence the use of LLINs. The mosquito trapping LLIN thus represents a tool with a high potential impact on the Global Fund's actions to combat malaria. Indeed, the primary functions of a LLIN are (i) to provide personal protection to the sleeper against mosquito bites, and (ii) to provide community protection through its mass-killing effect on the mosquito population. Obviously, a T-LLIN also fulfils these two functions, since it is first and foremost a LLIN. On top of that, the T-LLIN traps mosquitoes which then die, providing considerable added value. In the current study, almost half the mosquitoes killed by the T-LLIN were the result of trapping, and because of this mechanical function, the T-LLIN caused more mortality than other types of LLIN, including the 2nd generation LLINs currently advocated by the WHO in the event of insecticide resistance ^20, 21^. Considering this, the addition of a trap compartment to PN2.0 has led to a potential mass killing effect of 31.5% higher than that provided by IG2, 38% higher than that provided by Tsara boost (PBO-Net) and more than 58% higher than that provided by PN2.0, the 1st-generation mosquito bednet still widely used in African countries for malaria prevention. The increase in the mass killing effect will led to increased reduction in the density and/or longevity of mosquitoes resulting in community-wide protection that also benefits those who are not using bednets.

Another visible and measurable advantage of the addition of the trap compartment on the LLIN is the extension of the net's efficacy over time. Although we did not measure this factor due to the short duration of our study (6 weeks), it is well known that nets lose their insecticidal efficacy through washing and other exogenous factors ^22^. As the trap compartment acts mechanically, it is not subjected to the effect of time (except in the event of tearing) and should therefore prolong the lifetime of LLINs with huge positive impact on malaria transmission. In the present study, although we are in an insecticide-resistant area, the high mortality observed in the huts can be explained by the short duration of the study, since we used brand new nets (0–6 weeks old). This was confirmed by the WHO-cone tests on which we observed 100% mortality with IG2 and Tsara boost, and 58% with PN2.0 using mosquitoes from larval sampling of the local population. Therefore, an increase in the number of arms by including nets washed according to the WHO protocol would have enabled us to see the positive effect of the trap compartment on "used" LLINs.

One of the concerns of malaria prevention with LLINs remains the rate of use ^23^. As an advantage, we believe that the addition of a trap compartment to the LLIN can lead to an increase in the usage rate and even constitute an awareness-raising asset. Indeed, it's one thing to imagine that you're protected by a mosquito net, but knowing by the sight of mosquitoes trapped that you're protected is even greater. Entire awareness-raising messages on net use can be built around trapped mosquitoes.

Another concern of malaria vector control is insecticide resistance. The threat posed by this phenomenon to malaria prevention is well known ^2, 3^. According to the WHO, failure to mitigate the development of new insecticide resistance and manage existing ones can result in an increased burden of disease, potentially reversing some of the substantial gains made in controlling malaria over the last decade. Currently, resistance management is essentially based on delaying resistance genes development ^2^ only. The T-LLINs, in addition to delaying the emergence of resistance genes, can also act to limit their spread, as resistant mosquitoes that would have survived the lethal doses on the net and would have continue to spread the genes can be trapped and thus remove from the biological chain of reproduction. Consequently, by trapping resistant mosquitoes, the T-LLINs limit the spread of resistance genes and contribute to resistance management. Mathematical models can be used to estimate the impact of widespread community-wide use of T-LLIN on the spread of resistance genes. Use of insecticide-free T-Net would not select for any resistance whilst still protecting against malaria.

Although the cost of producing T-LLINs was not the subject of this study, the simple fact that manufacturing T-LLIN does not necessarily require the development of new insecticides (with uncertain efficacy) may considerably reduce production costs.

Taken together all these advantages, it is obvious that the T-LLIN should have a significant impact on malaria transmission, making it a tool with enormous potential for vector control, reinforcing the arsenal of existing products. Moreover, we used PermaNet 2.0 to build our T-LLIN within the framework of this study, though any brand of LLIN can be used, and the use of second-generation LLINs will achieve an even greater impact. The most important outcome of this study is that the principle or concept of mosquito trapping bednet which combine both a mechanical and insecticidal killing solutions works. Its effectiveness may vary according to the local context taking into account the diversity of insecticide resistance, but in any case, it can only be advantageous. According to recent WHO guidelines ^24^, for a candidate vector control product to be included in the new WHO intervention class without the need for epidemiological evidence, it must demonstrate non-inferiority to a first in class product which has already demonstrated public health value and superiority to a pyrethroid-only LLIN in experimental hut trials ^24^. The next step for us will be to contract an independent laboratory to carry out this evaluation study.

The current study shows potential to maximize the efficiency of the WHO-recommended LLINs by an addition of an insecticide-free trap compartment on top of the net. Because there is no insecticide on the trap compartment, there is no irritant and repellent effect and consequently, mosquitoes that are bouncing on top the net eventually enter the trap compartment regardless of their resistance status. The T-LLIN appeared to be more efficient in our study context than any other LLIN. In a wide-community use, the mass trapping of both resistant and susceptible mosquitoes is expected to reduce malaria vector populations in the long term and therefore lead to a decrease in malaria incidence and prevalence. Long-term use of T-LLINs is also expected to lower the spread of insecticide resistance genes. Acceptability and ownership of the T-LLINs should be greatly increased compared to conventional nets, given the real-life view of mosquitoes trapped each night.

## Ethics approval and consent to participate

The research proposal was approved by the National Ethics Committee for Health and Life Sciences of Côte d'Ivoire (CNESVS) N/ref:148-22/MSHPCMU/CNESVS-kp. Participants provided written informed consent.

## Data Availability

Zenodo: Trapping and killing performance of a PermaNet 2.0 hybrid trapping bednet: an experimental hut evaluation, https://doi.org/10.5281/zenodo.8150366 ^25^. This project contains the following underlying data: Crude data_Potential efficacy of a trapping LLIN.xlsx Data are available under the terms of Creative Commons Zero “No rights reserved” data waiver (CC0 1.0 Public domain dedication)
