# Supplementary material for: Trapping and killing performance of a PermaNet 2.0 hybrid mosquito trapping bednet: an experimental hut evaluation
Source: Wellcome Open Res. 2024 Mar 26;8:428. Originally published 2023 Oct 6. [Version 3] doi: 10.12688/wellcomeopenres.19759.3 (PMC10995533; doi:10.12688/wellcomeopenres.19759.3)
Supplement: Peer review history

# Reviewer report: Reviewer response for version 3

**DOI:** 10.21956/wellcomeopenres.23519.r104433

**Published:** 2024-10-23

**Authors:** Sulaiman S Ibrahim

**Affiliations:** 1 Liverpool School of Tropical Medicine, Bayero University, Liverpool, UK

**Overall:** This is an important manuscript which investigates the trapping and killing potentials of a PermaNet 2.0 LLIN mounted with a free-insecticide mosquito trapping compartment, which add a significant efficacy to this vector control tool. The manuscript is important and timely, as environmental, ecological, non-insecticide measures maybe the better arsenal for malaria control and elimination.

- But, the background needs to be updated, with the most recent situation with respect to malaria burden, with relevant references, and by addressing many claims which are only partly true.
- The methods need to be updated and corrected, and justifications should be provided for not providing prophylaxis to EH sleepers, and also for design contrary to the recommendation by the WHO.
- Results should be improved, in terms of text description and organisation and formatting and presentation of the figures. Most importantly, the other species (are they mosquitoes?) that authors did not describe at all.
- Discussion did not touch on other relevant studies across Africa in which EHTs were used to determine the efficacy of the major control tools. This should be captured broadly within the context of this finding.
- The manuscript will benefit from grammar corrections and contain many typos, some of which I have highlighted in bold in the respective sentences.
- Document line numbering should have helped to pinpoint where to make corrections, easily.

**Title**

 I suggest to modify the title of this paper into “ **Experimental hut evaluation of efficacy of a PermaNet 2.0-based hybrid mosquito trapping long lasting insecticide treated bed net**.” Efficacy can substitute for trapping and killing performance, avoiding the use of word ‘trapping’ twice in the title. Efficacy can be substituted with ‘effectiveness’ as well.

**Abstract**

 Background: WHO 2020 citation in the background is outdated. Please provide most recent details here, e.g., from the WMR 2022, or 2023.

 Results: Typing error in line 6. I think should read “…quantify the increased killing effect…”

 Line 8: Please put PN2.0 in full as PermaNet 2.0, unless if PN 2.0 is put first in bracket to indicate what it stands for.

 Conclusion: “…on top of the bed nets.”

**Background**

 Line 3: “impregnated in or impregnated onto…”

 “The susceptibility of mosquito vectors to insecticides determines the effectiveness of insecticide-based malaria control measures.” This is a weighty statement that needs to be referenced. And vector susceptibility is not the only factor that determine insecticide effectiveness. It is one of the major factors. This should be rephrased and relevant references should be provided please.

 The next sentence is the same. Reference should be provided, as this is not an opinion document.

 The sentence “To date, delaying the development of resistance to insecticides is the only resistance management strategy” is also only partly true, and should be rephrased and relevant reference should be provided.

 As advised in the abstract citing data from 2020 is not the best. Authors should please update their information  on the burden of malaria using the most recent World Malaria Report data and other relevant references.

 Third to the last line, please add a comma after the word, “innovative”

**Methods**

 Line 4: The word “in” is repeated. Please correct this.

 Line 7: *sl* should be s.l. as this is abbreviation

 “The experimental field station of Sakassou **is** located **about **500 meters from a large rice-growing area, and **comprised** 12 WHO-standard West-African type huts ^14^, **which were** slightly modified by removing/excluding the verandah **traps**.”

 Why did authors chose to rotate the bed nets after every three days, in contrast to the WHO recommendations? Authors should provide a reason within the manuscript to justify why they did this, while citing the relevant WHO 2013 document, or any more recent references.

 “… to Latin-square tables ^14^ **in** such a way that each bednet goes…”

 Please review this statement, as it is not clear and not helpful to non-expert. “…Prior to the trial, blank

 mosquito collection has been carried out to check for any hut’s effect.” Hut effect, define it and provide a reference.

 “Sleepers were all vaccinated against yellow fever and were taken to the hospital to check for malaria parasites.” Here, provide details of the YF vaccine. Also the sleepers should have been provided with malaria prophylaxis, for example doxycycline, or another more short-term prophylactic medicine, to protect them against malaria. This is an important ethical issue, an oversight which should have been taken into account, as it is a common knowledge in this field. It should be acknowledged in the methods and/or discussion.  

 Please correct the unit mg/m2 to mg/m ^2^. The number in superscript.

 “Tsara boost and IG2 are considered as WHO gold standards in insecticide-resistant areas in malaria vectors.” Provide a reference or a link for this statement.

 “However, since the T-LLIN we used **is made up partly of** a **deltamethri**n-treated section…” Also, please correct **deltametrin** to deltamethrin.

**Results**

 Line 2: An. gambiae s.l. as PCR identification to species level was not conducted. Please correct this throughout the document.

 “Among the other species, direct mortality observed with PN2.0 T-LLIN was 583 deaths out of 1150 mosquitoes collected, i.e. 50.7%.” This is not clear. What species of mosquitoes? Please provide breakdown with numbers and percentages of these species. There should be a figure, to provide composition of the different mosquito species caught. Please make this into Figure 1 panel a

 Figure 1: x axis need formatting with a border. Same with all other figures. The y axis should be provided as a percentage (daily mean mortality (%)), as is the usual convention. Please do the same to all other figures.

 All the figures 1-3 should be combined into one composite Figure, as panels b, c, d, with panel a being the figure I advised above. Combining this will aid better visualisation and comparison of treatment arms. In the end there should be two figures. The figure 1, with panels a, b, c and d; and Figure 2 (the current Figure 4).

 Figures should be presented after the first sentence describing them not contiguously as done by the authors.

 Figure 4: Please provide borders for y axis and make the y axis values to be 0-100.

**Discussion and Conclusion**

 Please correct Discussion/Conclusion to above, unless otherwise if this is what the F1000 requires.

 “The increase in the mass killing effect will **lead** to increased”

Is the work clearly and accurately presented and does it cite the current literature?

Partly

If applicable, is the statistical analysis and its interpretation appropriate?

Yes

Are all the source data underlying the results available to ensure full reproducibility?

Yes

Is the study design appropriate and is the work technically sound?

Partly

Are the conclusions drawn adequately supported by the results?

Yes

Are sufficient details of methods and analysis provided to allow replication by others?

Partly

Reviewer Expertise:

Vector Ecology and Biology, Impact of Climate Change on Health, Functional Genomics, Transcriptomics, Protein Biochemistry.

I confirm that I have read this submission and believe that I have an appropriate level of expertise to confirm that it is of an acceptable scientific standard, however I have significant reservations, as outlined above.

# Reviewer report: Reviewer response for version 3

**DOI:** 10.21956/wellcomeopenres.23519.r103522

**Published:** 2024-10-23

**Authors:** Gerry F Killeen

**Affiliations:** 1 School of Biological, Earth & Environmental Sciences and Environmental Research Institute, University College Cork, Cork, County Cork, Ireland

I’m very sorry to add the following substantive comment in the third round of review, especially for such an innovative and exciting piece of work, but this is the first time I’ve seen this manuscript and think the results are far too important to present inadequately. Despite the statistical shortcomings in the current version that I explain below, the overall conclusions look exciting, broadly correct and are unlikely to change following a more appropriate analysis using contemporary off-the-shelf statistical tools.

 These data can and should be analyzed with logistic generalized linear mixed models, which are now freely and easily accessible through R. This would allow not only proper means and confidence intervals to be estimated for the mortality rates, allowing for the controls, it would also enable estimation of nice odds ratios, also complete with 95% confidence intervals, for the contrast in mortality rates between the new T-Net and the existing alternatives. This approach, which allows repeated measures within units of observation like experimental huts over time to be accounted for with random effects, will also enable far more accurate estimation of p values in statistical contrasts than the archaic and inappropriate methods applied in the current draft, like Kruskal-Walis and Wilcoxon tests.

 Some elements of the sentence construction and use of terminology are a bit loose. For example, why use the term ITN after LLIN has already been defined and used several times?

Is the work clearly and accurately presented and does it cite the current literature?

Yes

If applicable, is the statistical analysis and its interpretation appropriate?

No

Are all the source data underlying the results available to ensure full reproducibility?

Yes

Is the study design appropriate and is the work technically sound?

Yes

Are the conclusions drawn adequately supported by the results?

Yes

Are sufficient details of methods and analysis provided to allow replication by others?

Yes

Reviewer Expertise:

Malaria vector biology and control

I confirm that I have read this submission and believe that I have an appropriate level of expertise to confirm that it is of an acceptable scientific standard, however I have significant reservations, as outlined above.

# Reviewer report: Reviewer response for version 3

**DOI:** 10.21956/wellcomeopenres.23519.r77722

**Published:** 2024-04-04

**Authors:** Armel Djènontin

**Affiliations:** 1 Université d'Abomey-Calavi, Abomey-Calavi, Benin

All comments from my review are addressed.

Is the work clearly and accurately presented and does it cite the current literature?

Yes

If applicable, is the statistical analysis and its interpretation appropriate?

Partly

Are all the source data underlying the results available to ensure full reproducibility?

Partly

Is the study design appropriate and is the work technically sound?

Yes

Are the conclusions drawn adequately supported by the results?

Yes

Are sufficient details of methods and analysis provided to allow replication by others?

Yes

Reviewer Expertise:

Medical Entomology, vectors control and insectcide insecticide management

I confirm that I have read this submission and believe that I have an appropriate level of expertise to confirm that it is of an acceptable scientific standard.

# Reviewer report: Reviewer response for version 2

**DOI:** 10.21956/wellcomeopenres.22901.r75233

**Published:** 2024-03-11

**Authors:** Armel Djènontin

**Affiliations:** 1 Université d'Abomey-Calavi, Abomey-Calavi, Benin

As part of the management of malaria vector resistance to various vector control tools, WHO recommends the research and development of new vector control tools. The authors set up a mosquito trapping system to manage resistance. The results obtained are well discussed and are in line with the methodology adopted. However, some concerns have to be addressed in order to improve the quality of the document.

**Abstract section**

 We suggest to authors to calculate additional killing effect of LLIN-t compared to LLIN and IG2 taking into account the negative control. The additional killing effect induced by the trap would be greater.

**Background section**

 Comment 1. ‘‘The two compartments being separated by the insecticide-treated roof of the lower compartment ‘’ or « The two compartments being separated by the insecticidal-free roof of the lower compartment »?

**Methods section**

 Comment 2: Give more details about the study area, rainfall, proximity to a rice or market garden, map of the study area.

 Comment 3: Why did you use 12 huts instead of 5? Given that 5 treatments were used.

 Comment 4: How many people slept in a hut each night? Explain how the 10 sleepers were used. To be more explicit, explain how you used 10 sleepers for 12 hut while an experimental set-up consisting of 5 treatments.

 Comment 5: According to the WHO (2013), for 5 treatments, a break of 2 days is required and the nets must be rotated after the break. In your study, why did you choose to rotate the nets every three days?

**Results**

 Comment 6: Review table 1: This table give the impression that there are 6 treatments.

 Comment 7: How do you explain the high mortality rate in the negative control? Before each rotation, did you wash the hut ?

 Comment 8: Uniform the graduation of figures

Is the work clearly and accurately presented and does it cite the current literature?

Yes

If applicable, is the statistical analysis and its interpretation appropriate?

Partly

Are all the source data underlying the results available to ensure full reproducibility?

Partly

Is the study design appropriate and is the work technically sound?

Yes

Are the conclusions drawn adequately supported by the results?

Yes

Are sufficient details of methods and analysis provided to allow replication by others?

Yes

Reviewer Expertise:

Medical Entomology, vectors control and insectcide insecticide management

I confirm that I have read this submission and believe that I have an appropriate level of expertise to confirm that it is of an acceptable scientific standard, however I have significant reservations, as outlined above.

# Response

**Published:** 2024-03-13

**Authors:** Mouhamadou Chouaibou Seidou

**Affiliations:** CSRS

**Abstract:** We have calculated and updated the Killing effect as suggested and updated the appropriate section of the manuscript with the new formula (Killing effect (%) = 100 x [(K _T-LLIN _– K _Un_) - (K _LLIN _– K _Un_)] / T _Un_,). **Comment 1: ** The right description of our trapping bednet is: “ The two compartments being separated by an insecticide-free or -treated roof of the lower compartment” This was corrected in the new version of the manuscript. **Comment 2:** “The average temperature in Sakassou is 26.1°C. Rainfall is recorded all year round, with an average annual precipitation of 899.6mm”. The method section has been updated accordingly. The experimental field station of Sakassou is located around 500 meters from a large rice-growing area. This detail has been added to the new version of the manuscript. However, we don't consider it necessary to add a map, as this is not a community intervention study with multiple study sites. We believe that the written description we have provided is sufficiently explicit about the station's location. **Comennt 3: ** We have 5 treatments, each composed of 2 replicates to make 10 huts. This has been clarified in the manuscript. **Comment 4:** We did not use 12 huts. This is a typo. We have mentioned 12 here only for the purposes of describing the resort. We used 10 huts. This has been clarified in the manuscript to avoid confusion. **Comment 5:** We have chosen to do rotation every three days because we were already replicating each treatment. **Comment 6 :** Done, data on trapped mosquitoes have been removed from the table. **Comment 7 :** Many factors can explain this, but overall we had 1.7 (less than 2 mosquitoes) deaths per day in the negative control hut, which is acceptable for such a study.

# Reviewer report: Reviewer response for version 2

**DOI:** 10.21956/wellcomeopenres.22901.r71985

**Published:** 2024-01-25

**Authors:** Benjamin Menze Djantio

**Affiliations:** 1 Centre for Research in Infectious Diseases (CRID), Yaoundé P.O. Box, Cameroon

The investigation carried out in this study was well done and demonstrated the efficiency of T-LLINs.

  WHO protocol was well followed.

 However, In the abstract background last line. The word only is not appropriate. we should see malaria control in an integrative manner combining all the methods available.

 It is also not clear to me why the author decided to use 12 huts and rotate the nets every 3 days instead of using 6 huts and rotating the nets every 6 days. In addition, the GLMM analysis should have given us a better understanding of the other parameters impacting the mortality observed. However, these observations do not reduce the high value of the work.

Is the work clearly and accurately presented and does it cite the current literature?

Yes

If applicable, is the statistical analysis and its interpretation appropriate?

Yes

Are all the source data underlying the results available to ensure full reproducibility?

Yes

Is the study design appropriate and is the work technically sound?

Yes

Are the conclusions drawn adequately supported by the results?

Yes

Are sufficient details of methods and analysis provided to allow replication by others?

Partly

Reviewer Expertise:

PhD in Vector Control Biology obtain at LSTM.  Head of the control tools unit at CRID and expert in Experimental Hut Trial

I confirm that I have read this submission and believe that I have an appropriate level of expertise to confirm that it is of an acceptable scientific standard.
